# The Social Origin of the Concept of Truth – How Statements Are Built on Disagreements

**DOI:** 10.3389/fpsyg.2020.00733

**Published:** 2020-04-28

**Authors:** Till Nikolaus von Heiseler

**Affiliations:** Institute of Philosophy, Freie Universität Berlin, Berlin, Germany

**Keywords:** storytelling, truth value, trophy display, meta-cognition, language evolution, naturalistic epistemology, indexical signaling

## Abstract

This paper proposes a social account for the origin of the truth value and the emergence of the first declarative sentence. Such a proposal is based on two assumptions. The first is known as the social intelligence hypothesis: that the cognitive evolution of humans is first and foremost an adaptation to social demands. The second is the function-first approach to explaining the evolution of traits: before a prototype of a new trait develops and the adaptation process begins, something already existing is used for a new purpose. Applied to the emergence of declarative sentences, this suggests something already existing—natural signs (which have a logical or causal relation to what they denote)—were used for the declarative function and thereby integrated (in the form of indexical objects implying a past action) into communication. I show that the display of an indexical object (such as the display of hunting trophies) can imply a conceptual structure similar to that informing the syntax of sentences. The view developed in this paper is broadly consistent with the argumentative theory of Mercier and Sperber, which suggests that reasoning is less adapted to decision making than to social purposes such as winning disputes or justifying one’s actions. In this paper I extend this view to the origin of the concept of truth. According to my proposal, the first declarative sentence (articulated in a simple sign language) emerged as a negation of a negation of an implicit statement expressed by the display of an indexical object referring to a past action. Thereby, I suggest that the binary structure of the truth value underlying any declarative sentence is founded on disagreements based on conflicts of interest. Thus, I deny that the *concept* of truth could have evolved for instrumental reasons such as solving problems, or through self-questioning about what one ought to believe.

## Introduction

### The Concept of Truth and the Ape Test

Essential aspects of human evolution—such as the development of language (Hauser et al., 2014), episodic memory (Suddendorf, 2013a) and mind reading (*theory of mind*) (Baron-Cohen, 1999)—are still not fully understood. One reason for this might be that human evolution is often misconceived. Though these distinctively human cognitive faculties have innate aspects—as shown by attempts to raise chimps as children (Kellogg, 1931)—they only develop fully through participation in a human society (Tomasello, 2019) as demonstrated by feral children (Goldin-Meadow, 1978). It has been convincingly argued that this ontogenetic fact points to a phylogenetic reality (Herrmann et al., 2007) and that most unique human cognitive faculties evolved mainly as adaptations to social challenges (Humphrey, 1976; Dunbar, 2011b).

In this paper I will suggest that this is also true of the cognitive ability to think in terms of true and false. The idea is that declarative sentences imply they are either true or false, and this binary structure is not a quality of the animal mind or of the natural environment but founded on epistemic disagreements based on conflicts of implicit interests. If this is the case, the truth value is—without being a contradiction—both of natural and of social origin.

The view presented in this essay extends Mercier and Sperber (2011) *Argumentative Theory of Reasoning*, the idea that reasoning evolved manly to help us win arguments in dialogic contexts or justify our actions (and not to pursue the truth), to the basic structure of a declarative sentence itself. At the same time, it is a contribution to the heterogenous and quickly growing field of language evolution theory (e.g., Bickerton, 2009; Fitch, 2010; Dunbar, 2011a; Tallerman and Gibson, 2013; Corballis, 2016; Everett, 2017; Cuskley et al., 2018).

Views on language evolution are divided over at least three questions: (a) whether language emerged for thinking, as inner language (I-language), or rather developed for communicative purposes, (b) whether there is a specific language faculty or language is rather built on general cognitive mechanisms, and (c) whether the language faculty evolved gradually or emerged suddenly in one step.

In this paper I shall defend the following view: (a) language evolved for communicative purposes—to transmit information about absent actions—not as I-language^1^. (b) There is a genetic foundation equipping human beings to acquire language (Tomasello, 2019), which includes various highly specialized talents such as the ability to understand syntax and symbols (Bickerton, 2005), including mimesis (Arbib, 2005), and that different individuals can have *contradicting beliefs* about a subject (such as where an object is hidden or whether an individual did something or not) (Hare et al., 2001). This implies grasping the distinction between representation and reality and is the cognitive basis for harboring the intention to be understood and to address the beliefs of others through speech acts. (c) I will suggest that the discontinuity of language evolution concerns *the functional aspect* of language (to transmit information about past actions influencing beliefs about the agents in a way that is beneficial to the genes of the communicator), while the language faculty—a widespread neutral network of highly specialized modules (Friederici, 2020)—evolved gradually, since the evolution of complex traits, corresponding to changes of allele frequencies in the gene pool, can only take place over a long period of time—although the evolution of the cognitive abilities of human beings has been remarkably rapid (Lahn et al., 2004).

I believe—as Chomsky (1965) and other theorists of generative grammar do—that syntax is essential to language, but also sympathize with theories of embodied cognition, which claim that higher cognitive functions, including language, are rooted in lower cognitive functions, such as the sensory-motor system (e.g., Arbib, 2005; Rizzolatti and Bucciono, 2005). This is to say, I shall embrace the situated cognition approach to the evolution of language while addressing the problem of the emergence of syntax as a necessary condition for the development of declarative sentences. This is done partly by adopting the *extended mind hypothesis* and applying it to syntax within the evolutionary process. Syntax, in this view, is not limited to relationships among arbitrary symbols (such as lexical items) but can include physical objects present in the communicative context (including the speaker) and their spatial and assumed causal relations. Likewise, as said before, in the evolutionary scenario suggested in this essay, the binary truth-value of propositions^2^ (which is the foundation of declarative sentences expressing them) is presented—though it might sound *prima facie* paradoxical—as an effect of the dispute over truth.

My hypothesis is consistent with Bickerton’s claims that for a trait as unique as language to evolve, the selective pressure that drove its development must also have been unique, because “otherwise the adaptation would have appeared elsewhere, at least in some rudimentary form.” (Bickerton, 2005, p. 514). This is why I reject the idea that language could have evolved for functions that we find in other animals such as trying to influence the behavior of present conspecifics (as accomplished in language by *directives*) or to express emotions (in language, *expressives*). This principle is sometimes called “the ape test” (Gärdenfors, 2013)^3^; expressed in normative terms, “Your hypothesis of language evolution needs to explain why the suggested evolutionary process only happened in our ancestors and not in other great apes, by suggesting a unique selective pressure for a new function.” Failing to meet this criterion of uniqueness is considered to be the main weakness of many (Hurford, 1999) if not all (Számadó and Szathmáry, 2006) theories of language evolution.

We shall see that my proposal is compatible with Dunbar’s suggestion (Dunbar, 2011a) that language is used for gossip to build trust as a foundation of reciprocity (though I do not believe that this was the initial function of language) and with Corballis’ idea (Corballis, 2016) that language evolved first in the form of sign language (though I do not believe that language, before being expressed, developed internally for mental time traveling).

### Evolutionary Models Used in This Paper

Which evolutionary model can be considered appropriate and safe to use depends on the context and purpose. Most discussions in evolutionary theory—such as the debates about epigenetic phenomena (e.g., Banta and Richards, 2018; Lucchesi, 2019), neurological plasticity (e.g., Blumberg et al., 2009), gene expression (Taylor et al., 2019), gene–culture coevolution (e.g., Gintis, 2011), alternative models of inheritance (e.g., Jablonka and Lamb, 2008) and multi-level-selection (e.g., Wilson, 2006; Wilson and Wilson, 2008)—are not about replacing the concept of natural selection, but whether we need to extend the *standard model* to explain human cognition and its development from that of a fertilized egg to that of a mature adult. In contrast, in this paper I merely aim to explain the inherited basis of a trait (and neither the ontogenetic development nor how the trait actually works in everyday life). In this case, it seems to be safest only to use general and widely shared assumptions about evolution. Every evolutionary development you can explain with the *standard theory* you can explain with extended versions, but not necessarily vice versa (Pigliucci and Müller, 2010).

The function-first approach suggested in this paper is compatible with *standard models* of evolution, including Darwin’s account of natural selection (Darwin, 1859), the modern synthesis (Fisher, 1930; Dobzhansky, 1937), the *inclusive fitness model* (Hamilton, 1964) (Maynard Smith, 1964; Williams, 1966) and with most, if not all, extended evolutionary models [such as that suggested by Pigliucci and Müller (2010)]. Please note that this is no statement on ongoing discussions on evolution, but simply a strategy for not engaging in those debates.

### An Outline of Three Problems for Language Evolution

Regarding language evolution there are at least three major questions. The first two are fundamental to any evolutionary history and originally addressed by Tinbergen’s questions about the two *ultimate causes*: function and evolutionary process (Tinbergen, 1963), while the third is specific to language.

(1) *The function.* What did the trait evolve for? Here the challenge is *to suggest the right kind of selective pressure* for the trait to develop. Language, for instance, could not have evolved for merely transmitting valuable information, because this would give a reproductive advantage primarily to the receiver (and not to the communicator). Also, it seems unlikely that language could have evolved for better coordination of groups, for two reasons: firstly, most coordination concerning the here and now can be done without language, and secondly, better group coordination gives advantage to the group, and no complex trait can evolve by group selection (Williams, 1966). As already mentioned, we need to ascertain not just why our ancestors evolved language, but also why other great apes did not, which would suggest a unique selective pressure for a new function (Gärdenfors, 2013). In an ideal evolutionary scenario, a slightly better speaker would out-reproduce all others.

(2) *The evolutionary process.* How did the trait begin to develop and then evolve *gradually*? This relates to the challenge of *irreducible complexity*, first introduced by opponents of evolutionary theory (Behe, 1996) and then turned into the following challenge: traits can only evolve if they have a function, but in some cases, the function already demands a complex structure (e.g., when the structure consists of well-matched interacting parts of which none has a function on its own).

I will discuss this issue regarding declarative sentences with respect to syntax in the subsection *Irreducible complexity I: syntax* and with respect to truth values in the subsection *Irreducible complexity II: the truth value.* Why both require explanation will be clarified in the course of the argument.

(3) The third problem is specific to language evolution and concerns the problem of cooperation. I will address this issue in the next subsection.

### The Evolution of the Cooperative Principle

As already noted, language probably did not evolve for transmitting valuable information to non-kin [as suggested by Pinker (1995) and Pinker and Jackendoff (2005)] because this would give the receiver a reproductive advantage over the sender (Fitch, 2004). There is another widely discussed problem with language evolution: language is unreliable (Zahavi, 1993), because there is no natural connection between signifier and signified (Saussure, 1916), and speech is cheap (Maynard Smith, 1994) compared to costly and thus hard-to-fake signals such as the train of a peacock (Zahavi, 1975). Therefore, it needs to be explained why our ancestors began trusting each other’s linguistic claims (Power, 1998). Thus, many researchers have said that before language could even begin to evolve, a kind of *cooperative principle* (Grice, 1975) had to be in place (e.g., Wacewicz and Żywiczyński, 2018; Ferretti et al., 2018). However, this just seems to postpone the problem of language evolution to the problem of the emergence of cooperative behavior, which also appears to be unsolved (Alexander, 2008).

Now, researchers who work on the problems of altruism and cooperative behavior from a game theoretical perspective argue that human altruism is based on gossip (Barclay, 2010). If gossip circulates in a group and reputation is based on what other people say about you, then it seems rational to develop cooperative behavior (Alexander, 1987; Krebs, 1998; Sperber and Baumard, 2012); in other words, in this view, reputation must be at stake all the time (Lerner and Tetlock, 2003).

Here is the paradox that emerges when we put both explanations together: on one hand, it requires language for gossip to circulate as the basis for cooperative behavior, and on the other hand, it was said that for language evolution even to start, individuals have to cooperate by communicating truthfully, since this is the foundation of most linguistic exchange [cf. (Grice, 1975)]. Accordingly Bickerton and Szathmáry (2011) wrote that one main demerit of most approaches to language evolution is that the evolution of cooperation and the evolution of language are treated separately. How a consistent theory addresses both is the litmus test for any suggested hypothesis.

### Irreducible Complexity I: Syntax

The structure of even the simplest declarative sentence seems too complex to spring into existence from nowhere. To express most propositions (including those referring to non-present actions) requires a minimum of a verb and the arguments required by the valency of that verb. A structure cannot exist without its elements. Thus, most people believe that concepts (Chomsky, 1966) or words (Pinker and Jackendoff, 2004) must have existed before sentences were thought or expressed. The problem: one element of the structure—the verb—cannot be tangibly thought or expressed without alluding to its implied syntactical structure. But then again, this syntactic structure (how the verb selects its arguments and complements) cannot exist without its elements.

Our suggestion for solving this paradox will be that the first verbs (signs referring to actions) were not part of a symbolic composition (of e.g., lexicon items) but rather embedded into a constellation of physical objects: a quasi-syntactical structure emerges between entities in a physical space in a concrete situation while the verb is expressed in the form of a *directed mimetic gesture* marking the entities with their thematic roles. We will discuss how this works in more detail later.

### Irreducible Complexity II: The Truth Value

Understanding declarative sentences includes understanding the binary logical form of the truth value, because to understand a declaration means to understand on what grounds it could be denied (Williams, 2004). If one expresses a declaration (S1) one could always add “S1 is true” (S2); this is to say “It is true that S1” is logically equivalent to S1 itself (Copi et al., 2014).

Here is the problem in terms of evolution: on one hand “S1 is true” (S2) is *implied* by S1: without S2 no S1. On the other hand, a truth value (S2) might be seen as a kind of meta-predicate commenting on S1; a statement that has another statement (S1) as its subject. Here S1 is a necessary condition of S2: without S1 no S2. What could be considered a logical equivalence creates in an evolutionary context a c*hicken–egg dilemma*: if S1 (any declarative sentence) cannot exist without S2 (its truth value), and S2 not without S1, both have to emerge at the same time. Additionally, abstract binarity did not exist in the perceptible natural environment of our pre-human ancestors (negation does not exist in nature). It is also doubtful that they were born with a concept of binarity in their minds. I will try to convince the reader that the binary value of truth has a social origin, stemming from conflicts of interest, and that an explicit declaration could emerge in the context of a social interaction as a *negation of a negation* (based on the social activity of mutual rejection). We will see that this implies that meta-semantic cognition (understanding the beliefs of another individual) preceded the first declaration.

So far, we have discussed three problems that need to be solved by any theory of language evolution. In the next subsection I introduce the concept of an *evolutionary turning point* (ETP). We will define an ETP as the point where a function is positively selected for, for the first time. To find the ETP of language, it is essential to understand how language evolution began.

## Evolutionary Turning Points

### Chomsky and the Problem of Preadaptation

Several scholars have claimed that Chomsky’s concept of language is incompatible with evolution (e.g., Bickerton, 2010; Ferretti et al., 2018). I think this is true, but for a slightly different reason than generally presumed. Chomsky conceptualizes language as a “computational system of the mind/brain that generates an infinite array of hierarchically structured expression” (Chomsky, 2010, p. 45). He argues that a limited and inflexible system, such as that of animal calls, cannot develop gradually into the wonder machine of language production and suggests that this change to *infinite productivity* depends on a single irreducible innovation: *recursion* (Chomsky, 1956), which he describes, in the framework of his *minimalist program* (Chomsky, 1993), as unbounded *merge*: “an indispensable operation of a recursive system […] which takes two syntactic objects A and B and forms the new object G = {A,B}” (Chomsky, 1999, p. 2), rereading itself. According to this line of reasoning the mental revolution of human evolution depended on a single new ability in cognition:

Within some small group from which we are all descended, a rewiring of the brain took place in some individual, call him Prometheus, yielding the operation of unbounded Merge, applying to concepts with intricate (and little understood) properties (Chomsky, 2010, p. 59).

In other words, Chomsky suggests, a single mutation could have changed the relations between *already existing concepts* by introducing a single operation—merge—in a recursive way (results of past merge can be objects of future merge). This model of serial processing is embedded in the neo-Cartesian intuition (Chomsky, 1966) that language-like thought precedes linguistic expression (Chomsky, 2006).

In this view language emerges as I-language, which later becomes externalized. Here the mind is conceptualized basically as a box, with no hands, which is not situated in a physical environment or in a network of social interactions [for critique cf. (Clark, 2016)]; as a consequence it can only use internal elements in its computation and could not integrate physically present objects as symbols into a syntactic structure (as is easily done, for instance, by users of sign languages)^4^. However, the main problem concerns Chomsky’s concept of biological development: evolutionary processes are unlikely to begin with a mutation or even with a new behavior caused by a mutation alone. On the contrary, such processes begin with an organism introducing a new strategy more or less by chance under given circumstances.

Most scholars would agree that some organic structures evolved because they improved the reproductive fitness of organisms by fulfilling a function (Williams, 1966): the eye to see, the ear to hear, and the legs to walk^5^.

Now, since the structure of the ear making hearing possible is an adaptation to the function of hearing, how can this process even start? The wrong answer would be to say with a mutation that suddenly made hearing possible. A better idea is that something that evolved for other reasons is used for a new purpose. In these cases, the older structure is *preadapted* to the new use (Darwin, 1859). Preadaptation has been misinterpreted in teleological terms (Gould and Vrba, 1982) suggesting it would designate an *adaptation before the evolutionary process started* (which would be paradoxical), but Darwin just meant *a structure that can be used for a new function before it is adapted to a new function in the evolutionary process by use.* Thus, *preadaptation* refers to a given structure adopting a new function with little or no biological modification (Ardila, 2016). I will illustrate what this means with an example in the next subsection.

### The Function-First Approach to Explaining the Evolution of Traits

One of the most impressive and well-studied transformations in evolutionary biology is the transition of fish to tetrapods, the adaptation of the locomotion that made the conquest of land possible, which happened about 400 million years ago (Janvier and Clément, 2010; Niedźwiedzki et al., 2010)^6^. It has been thought that fish were washed ashore and then crawled or leaped, using their fins; and because of this use, the fins slowly evolved into legs. More recent research (Clark et al., 1994) shows that a four-legged gait had already developed in water and that these fish even had fingers and toes.

**FIGURE 1:**
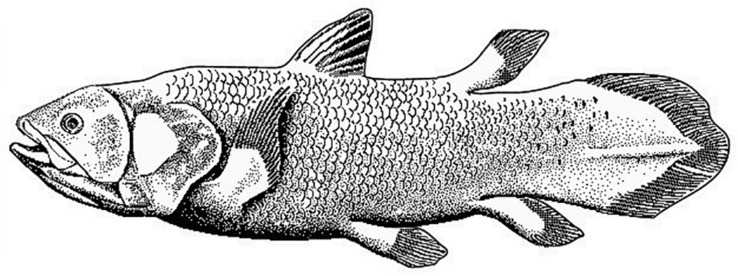
Sarcopterygii.

The crucial point in the evolutionary development of four-footed ambulation was not the movement from water to land but the point at which an individual could improve its reproductive success by using its fins as legs in the water to walk on the bottom of the ocean. If we could have been observers of this behavior, we might not have been very impressed by it. Nevertheless, this line became extraordinarily successful, since all terrestrial vertebrates including amphibians, reptiles, birds, and mammals are descended from this fish using an older structure (the fins) for a new purpose (walking). At the beginning of this extraordinary success story, some fish achieved a reproductive advantage over their peers by introducing a new strategy. From this moment on there was a more or less continuous development of four-legged locomotion until it found the optimal mechanism in each land animal. But the crucial point in this development was the moment when a particular bony fish—the *Sarcopterygii* (Figure 1)—used its fins as legs and thereby obtained a reproductive advantage.

If we take this as an analogy for the development of language, the incredible success of land animals is comparable with the omnipresence of linguistic culture. The most important lesson we can learn from this example is: the *evolutionary turning point* (ETP) is not a mutation but *a complex interaction with the environment in a specific situation that produces a reproductive advantage.* Maybe the first walking behavior occurred when a fish hid from a predator between plants at the bottom of the ocean and used its fins touching the ground to creep under a plant without creating the motions in the water that its predator was evolved to detect. Being a beneficial behavior depends on the circumstances and environment—in this case the predator (including its specific abilities for detecting prey), the plants, and the contingent behavior of using the fins as legs. If a mutation played a role in making one individual more likely to use something for a new purpose, then this mutation would concern the inclination to behave in a certain way in a given situation rather than a modification of the body shape. Even if a fairy were to magically give fish legs, the evolutionary turning point would occur only when they were put into use in a specific situation in which they would produce a reproductive advantage.

**FIGURE 2:**
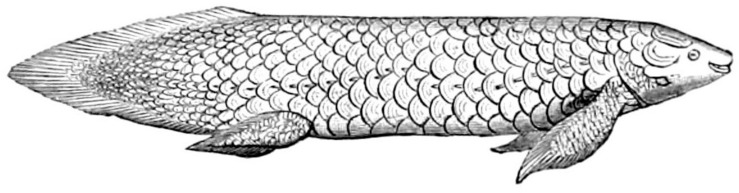
Queensland lungfish (showing some adaptations to walking).

After this turning point, traits develop (Figure 2) depending on various factors including selective pressures, genetic variation in the population, and the frequency of occurrence of mutations beneficial for the trait and the size of the population. Over the course of this adaptation, every little change in structure must increase the reproductive success of the individuals (Mayr, 2001). Thus, while a discontinuity occurs at the system level with the first appearance of a new function, the species can evolve only gradually over generations (which can be, in certain circumstances—for instance in the process of speciation—faster than traditionally thought Gould and Eldredge, 1972; Newman and Bhat, 2009).

Now, if the *evolutionary turning point* is the point at which a function is fulfilled for the first time, creating a reproductive advantage, there are no structures already adapted to fulfill the function of the future trait. Consequently, *something* (x) that evolved for other reasons is used at the evolutionary turning point for the new purpose—the function the trait will fulfill. Traits can evolve only for functions that cannot be as effectively fulfilled by already existing traits. The *original evolutionary function* (OEF) of the trait is the function a trait originally develops for. The OEF creates a prototype (p) of the trait that fulfills the new function in a simple way. A good evolutionary explanation unveils the original function (OEF) of a trait, reconstructs the prototype (p) and identifies the structure the prototype developed from (x). This leaves us with three questions:

(α) What is the first and cognitively simplest form of a declarative sentence? (p)
(β) Why was it reproductively beneficial to transmit propositional content? (OEF)
(γ) What is the structure that emerged originally for other reasons that could have been used to transmit propositional content before language existed? (x)

## What Are the Cognitively Simplest Linguistic Expressions?

In the last section I presented the function-first approach: the evolutionary process starts with putting something already extant (x) to a new purpose (OEF). I also introduced the concept of an *evolutionary turning point* (ETP), which we shall find useful in later discussion. In this section I will reevaluate the relevance of *declarations* in the context of language evolution (that language evolved for fulfilling a declarative function); then I shall answer the first question (α) by identifying *p*, which will suggest the identity of x (the older structure used for a new purpose) and specify the *original evolutionary function* (OEF).

Traits, when fully developed, often serve diverse functions. With my ears I can feel the temperature and wind, hear the sounds of my natural environment, listen to speech and symphonies, and keep my glasses on my head. To find the OEF of a trait or an organ we have to consider, as noted before, the aspect of it that cannot be fulfilled as effectively by other traits or organs. I can feel the temperature with my ears, but I can also feel the temperature with other parts of my body. This makes it unlikely that my ears actually evolved for temperature detection. The same would be true with regard to the use of language to issue commands or imperatives and express exclamations (Bickerton and Szathmáry, 2011; Gärdenfors, 2013).

There is a growing literature on the communication of chimpanzees. It was found that wild chimpanzees use several dozen gestures to communicate and all of them fulfill an imperative function (Hobaiter and Byrne, 2014). Most animal species with parental care also express pain and fear through exclamations. According to a long tradition going back to classical Greek grammars, there are two more functional categories of sentences beside imperatives and exclamations: *declarations* and *questions*^7^. Both are unique to humans^8^. It seems unlikely that language evolved for asking questions, because to ask questions only makes sense when someone can answer. This suggests that language indeed evolved originally for the function that declarations—but not other classes of speech act—can fulfill.

Most theorists agree that declarative sentences fulfill the function of transmitting propositions (Akmajian, 1984). If this is true, it is likely that the *original evolutionary function* of language can be further specified by reconstructing the most primitive relevant form (p) of such transmission. Now, what are the minimal conditions for fulfilling the declarative function in the cognitively simplest way?^9^ Note that cognitive simplicity is not an intrinsic quality of a structure itself but a relation between the structure to be understood and a cognitive system, which is evolutionarily adapted for certain types of cognition in certain contexts. This is why cognitive simplicity might not be identical with technical or logical simplicity.

(1) The simplest syntactic structure that can fulfill the declarative function consists of a verb and its arguments (*n*-valent verb and *n* arguments) (Tesnière, 2015/1966). The technically simplest sentence would have as few elements as possible, consisting of one verb and one argument representing the subject. This would make “Jill grows” simpler than “Jill hits Jack.” However, there is evidence that non-human primates understand only *transitive actions* (actions involving other animals or objects (Rizzolatti and Arbib, 1998; Rizzolatti and Bucciono, 2005).

**FIGURE 3:**
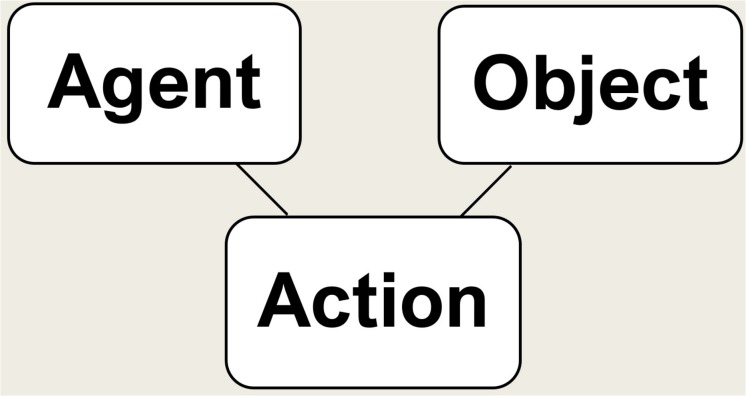
The cognitively simplest declarative sentence.

Therefore, the cognitively simplest syntactic structure corresponding to a pre-linguistic primate conceptual structure includes not only a verb and an actor, but also an *object* (a syntactic category which may play a variety of conceptual roles, such as *patient* or *theme*). Consequently, the simplest transitive verbs are *bivalent* with two semantic argument slots (agent, patient)^10^. This would make the cognitively simplest structure look like this (Figure 3). The object could be for instance a physical object or another animal, including a conspecific—typically something changed, addressed, targeted, or manipulated by the action—comparable to the thematic role of a *theme* or *patient*.

(2) Singular terms (expressions that designate physical objects—including animals and conspecifics) are cognitively and communicatively simpler than general terms (categories), since it is possible to point to the reference of a singular term, but not to the reference of general terms (Frege, 1892).

(3) The simplest tenses are the present and the past because the concept of the factual is simpler than the counterfactual (future or imaginary scenarios), since counterfactual scenarios need the capacity to distinguish between reality and representation. To draw attention to an overlooked present (such as a hidden predator that is about to attack) requires no syntactic structure and can be accomplished by pointing or with an indexical call (e.g. a alarm call), such as are already part of some animal communication systems. In other words, only reference to some non-present actions requires the essential features of human language (such as syntax and symbols), and therefore these constitute the simplest relevant scenarios. Non-present events are easier to represent if they are fresh in memory.

(4) The simplest *aspect* (how actions, states or events extend over time) is that of a single incident at some point in time (Verkuyl et al., 2006).

(5) Verbs that express observable actions seem cognitively simpler and easier to communicate than abstract verbs. “Jill hit Jack” is simpler than “Jill intimidated Jack.” (Számadó and Szathmáry, 2006).

Consequently, we must conclude that the simplest relevant declarative sentence is about a *concrete* (formerly observable) *past single* event including an *agent* and a *patient*.

(6) The simplest grammatical person to express (and to understand) seems to be the first-person singular, which is always present and thus most easily indicated or implied. In sign languages first-person sentences can be expressed without role-taking, whereas other grammatical persons require perspective switches [between the verb—signed by the speaker—and the grammatical person (Janzen, 2017)].

(7) Mimetic gestures are less demanding than conventional ones (Peirce, 1931; Deacon, 1997).

(8) In most sign languages, a sentence can be expressed by including the sender and the receiver as symbols in the syntactic structure of the sentences expressed, marking them with their thematic roles relative to the verb; “I give you the book” can be, for instance, expressed with only one gesture directed from the speaker to the receiver while shaping the hand in a way that signifies a book.

**FIGURE 4:**
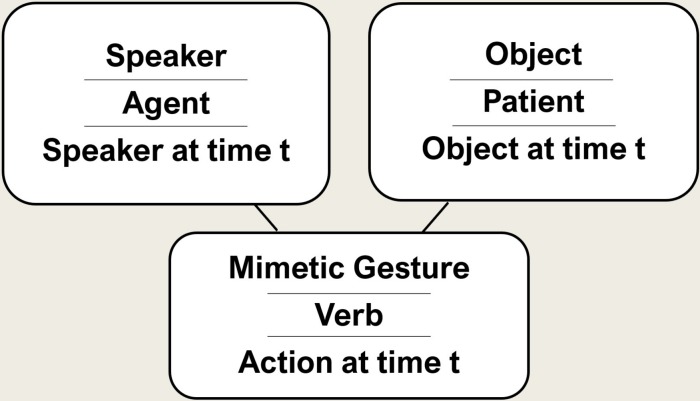
Primitive Sentence: speaker symbolizes the agent, a present object the patient and a mimetic gesture the verb. The agent signifies the agent in the past (when he did the action), the patient the object in the past (when the action happened) and the verb the action.

Arguments 1–8 can be brought together in the following way. The simplest relevant declarative sentence refers to a concrete past single transitive action consisting of three elements: (a) the sender as the agent, (b) a present object (a living being or an inanimate object) as the patient or theme, and (c) a directed mimetic sign—representing the action—one that marks the agent and the patient in their semantic roles (Figure 4). This would make the first declaration a minimal narrative about oneself, with the properties described above.

We have thereby answered the first question: (α) What is the first and cognitively simplest form of a declarative sentence? With this primitive sentence in mind we can modify the last two questions we asked at the end of section 2: (β) Why was it reproductively beneficial to transmit propositional content? (γ) What is the structure that originally emerged for other reasons that could have been used to transmit propositional content before language existed?

(β′) How is it reproductively beneficial to refer to one’s own past actions? (OEF)
(γ′) What is the structure that could be used to refer to one’s own past actions before language existed? (x)

To refer to one’s own past could be beneficial to the communicator for many reasons influencing social alliances, social hierarchy, and both directly and indirectly (e.g., via hierarchy) the sexual selection of potential mates. In all these cases communicators influence beliefs about themselves in a favorable way.

As already mentioned, the ETP is a product of a complex interaction between the organism and the environment in which many different factors such as genetic disposition, behavior, circumstances, and coincidence play a role. At the ETP an individual acquires a reproductive advantage with regard to a new function for the first time that eventually will develop into a trait. This is, as we shall see in the next section, particularly important in relation to the problem of the emergence of language, because early language use is always embedded in a social context in which there is at least a sender and a recipient present.

In this section we identified the cognitively simplest declaration as a statement about one’s own past actions. Nevertheless, to suggest that such statements constituted the first linguistic actions seems to go against a deeply ingrained intuition embedded in the misconception of primate evolution being constituted from adaptations for the survival of individuals or a group in certain environments. Based on this instrumental conception of primate cognition, one might assume that to talk about the future would be more adaptive than referring to the past (Gärdenfors, 2013).

However, there are at least the following six arguments supporting the hypothesis that language first evolved for referring to the past rather than the future or present: (1) Even complex forms of group coordination do not require linguistic communication—as shown by hunting chimpanzees (Mitani and Watts, 2001); (2) Most simple teaching can be done by demonstration and does not require language (e.g., people who don’t know sign language showing deaf children how to cook or how to repair a bike without using conventional language); (3) Simple forms of planning and instruction often use only imperatives; (4) To say something about the future could transmit information which would benefit the receivers rather than the sender (cf. Introduction). (5) Even if, for the sake of argument, teaching and planning might use mimetic gestures, this would not explain the evolution of the cognition of truth values. Like all claims about the future, planning does not produce a divergence between representation and reality [cf. (Austin, 1962)]. (6) Primate evolution is driven mainly by social—sometimes described as Machiavellian (Byrne and Whiten, 1988)—intelligence, which is expressed in *political* behavior that aims at superior reproductive success for the competitor (de Waal, 1982). All these claims are not necessary for the argument presented in this paper but are only mentioned to provide further support.

In the next section we discuss *how* one might have referred to one’s own past actions before language existed—in a world with no symbolic communication, no understanding of pantomime and mimesis, and maybe not even the intention to transmit propositional content.

## Declarations Before Language

### Referring to Absent Actions

We said that the precursor of a trait should be something (x) that can fulfill the purpose of the later trait without being adapted to that function beforehand. At the evolutionary turning point of language evolution, something which already existed must have been used for the OEF of language. We suggested that the OEF of language is to transmit information about a concrete past action of the sender, with results beneficial to the reproduction of the sender’s genes. Thus, the question arises of how one can refer to one one’s own past actions without language—no symbol use, no comprehension of conventional or mimetic signs, not even the simplest form of mimesis, pantomime.

One thing we can say with certainty: only signs can refer to anything. If everything that refers to something is a sign (more precisely, is processed in a given context by someone or something as a sign for something), only signs can refer to past events. We can distinguish two (mutually exclusive and jointly exhaustive) classes of signs: *natural* and *non–natural signs* (Grice, 1957). The latter include mimetic and conventional symbols and are expressed intentionally. Natural signs, in contrast, have no author, do not use mimetic^11^ or conventional symbols, and come into being without intention. They are causally related to what they designate, such as the smell of a rose and the shadow of a wolf’s head in moonlight. All natural signs are indexical (while not all indexical signs are natural). In a world in which only natural signs exist, thus only indexical signs can refer to past events. Indexical signs referring to past events include, for instance, tracks of animals indicating the animals’ earlier passage and the remains of a zebra on which a lion gnaws indicating its past kill.

This would suggest that at the ETP of language evolution *indexical signs* were used for the OEF of language: to refer to a past action of the sender. Following the function-first approach to explaining evolution, these natural indexes referring to past actions of the sender would be integrated into communication and *this communication would develop into language.* To relate this hypothesis to our more concrete analogy, concerning the ETP of walking, the *indexical signs* are analogous to the *fins; referring to one’s own past* is analogous to *walking*; and *integrating those indexical signs referring to the sender’s past into communication* is analogous to *using fins for walking*.

### Indexical Signaling

One criterion for eligible evolutionary scenarios in which indexical signaling could have become declarative is that they should not presuppose the cognitive abilities whose evolution they want to explain. In other words, the scenario needs to be possible without any syntactic competence, without comprehension of symbols, and without imitation. In the very beginning there might be—and this may sound paradoxical—no intention to communicate, and perhaps not even a memory of the communicated past action of the communicator and no conscious understanding on the side of the receiver. If an indexical signal is accurate and evolutionary beneficial it connects a past action with reproductive benefits, even if neither the sender nor the receiver has conscious representations of the past action [cf. (von Heiseler, 2019)]. Nevertheless, a display behavior would have emerged under this circumstance if it improves to affect the minds of the audience and influencing their behavior in favor of the sender’s reproduction. These *mental states* must produce a behavior as if the organism were to have a belief^12^.

Although the function-first theoretical approach would assume that linguistic communication developed from a behavior affecting (not necessarily intentionally) the mental states of a conspecific, there is no reason to believe that most signals that affect the mental states of other animals have any tendency of developing into anything more. Actually, affecting the mental states of other animals is widespread in the animal kingdom [cf. (Dawkins and Krebs, 1978)], from bird song and expensive signaling in sexual selection to the fake broken wing displays of the piping plover (a bird that feigns a broken wing to lead predators away from the nest), to mimicry. This suggests there is something peculiar about indexical signaling of *a past action of the sender* that qualifies the particular behavior to become the forerunner of simple language use.

One possible categorization of indexical signaling follows the classification of the object which was changed, addressed, targeted, or manipulated in the past communicated action, typically the patient. Patients can either be living creatures—for instance conspecifics in a social situation—or inanimate objects. Such inanimate objects we shall define as *trophies*. In the next two subsections, I will explore the trophy display scenario; in the following two subsections I will discuss indexical signaling that includes other animals such as conspecifics, and the significance of their reactions.

### Trophy Display

We will define a trophy as an object that indicates that the presenter performed an action that would be beneficial for them to communicate. This wide definition would not only include hunting and war trophies, but also crafted artifacts, rare objects or any object that is hard to acquire or to defend.

For this to work as an evolutionary scenario, trophy display needs to be somehow reliable and selective; this is to say, individuals of a group must differ in their abilities to acquire and display trophies, and that must produce differences in terms of reproductive fitness. The cost of acquiring such a trophy might include putting oneself in physical jeopardy or could be costly for other reasons. In such a case it would be a form of costly—and thereby unforgeable—signaling, which is a common object of female mate choice^13^ in the natural world (Zahavi, 1975). The difficulty of acquiring the trophy would correlate here with its rareness (selective function). An indexical object could for instance show the presenter’s cognitive abilities, courage, physical strength, skills, or other exceptional abilities and personal qualities.

If trophies were the objects of direct or indirect sexual selection, the targets of sexual selection could easily expand from directly observed qualities and manifest behavior (situations without indexical signaling) to any behavior reliably indicated by an indexical object. This would improve the quality of sexual selection immensely (which benefits the respective choosing sex).

Furthermore, the abilities to acquire such indexical objects should be put under selective pressure as demand increases in a feedback loop of competition. By adopting a rather wide definition of a trophy, many traits could be put under selective pressure when trophies are positively selected for: all the technical skills required to obtain a trophy, including the production and use of weapons, and forms of collaboration in hunting or head-hunting, but also the production of artifacts that make the owner more desirable by mates or which could improve their social status.

In the beginning of the described process, as already noted, neither the displayer nor the receiver needs to be conscious of the action the trophy implies. Since trophies are expensive to acquire and the presentation of them is cheap but limited by cognition, the cognitive aspects that limit the display behavior or could improve it should be put under strong selective pressure. Here the recognition of the attention of the relevant others might develop because being aware of the attention could modify the display behavior in a beneficial way.

### Trophy Display as the Missing Link

Maynard Smith and Szathmáry (1995) discussed *major transitions in evolution* by reconstructing possible pathways crossing the gap in question. Applying this method to trophy display, we shall concentrate on two gaps: (1) the gap between ape behavior and trophy display and (2) how trophy display could transform into a prototype of linguistic behavior.

(1) *Indexical signaling in apes.* The simplest form of trophy display would be to carry around an object causally related to a past action. If it would be beneficial for the receivers to modify their behavior in a way that would be beneficial for the displayer, this would be enough for the selective process to begin. The first competence that should evolve in such a situation on the side of the sender is an index display behavior, a behavior that draws the attention of the relevant others to the indexical sign (without necessarily the intention to communicate).

As an example of such a behavior, male^14^ chimpanzees carry around corpses of infants they have killed from another group (Goodall, 1977) or other killed animals without eating them (Hirata et al., 2001; Carvalho et al., 2010). This interaction with dead bodies (which in most cases has been misinterpreted as *play* or *investigation*) could be an instinctive technique for drawing attention to the trophy even before the comprehension of the attention of the other has been developed, let alone understanding the mind of the other or any concept of intentional communication. This behavior could evolve if the receivers modify their behavior in favor of the trophy displayer, for instance if the behavior is considered a warning against challenging the displayer (although the bodies displayed would rarely be chimpanzees of the group in question) or if the behavior makes the displayers more attractive for reciprocal exchanges or to potential mates.

**FIGURE 5:**
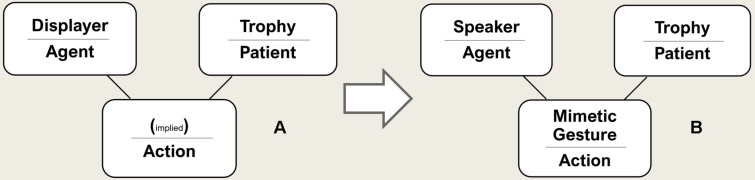
The transition to language. Left the structure of trophy display **(A)**; Right the structure of the simplest sentence **(B)** (cf. section “What are the Cognitively Simplest Linguistic Expressions?”).

(2) *From trophy display to a prototype of linguistic behavior.* When someone presents the skull or horns of a buffalo or the head or heart of an enemy, this could be interpreted as declaring “I killed this buffalo or enemy!” The presenter might be understood as an agent, the trophy as referring to the patient, and the state of the trophy as implying the concept of “killing” (Left side of Figure 5, A).

If now a single mimetic gesture were added interrelating these elements, a declaration is expressed, including the sender and a present object as symbols of themselves in the way sign language today incorporates present persons and objects into syntactic structures (Right side of Figure 5, B).

However, a problem arises concerning the occurrence of the first mimetic gestures: Either a trophy presentation is understood by itself, or it is not understood. If it is understood—by provoking an inner representation of a past event—adding a mimetic gesture seems unnecessary. If the trophy display is not understood, the invention of a first mimetic gesture in a world where no mimesis is in use would be unlikely to help understanding either. In either case, adding a mimetic gesture to a trophy display should not contribute to the comprehension. In this subsection I presented trophy display (Table 1, column 3); in the next subsection I will discuss indexical constellations, in which the indexical signal includes conspecifics (Table 1, column 2).

**TABLE 1:** Three stages of transmitting information about one’s past.

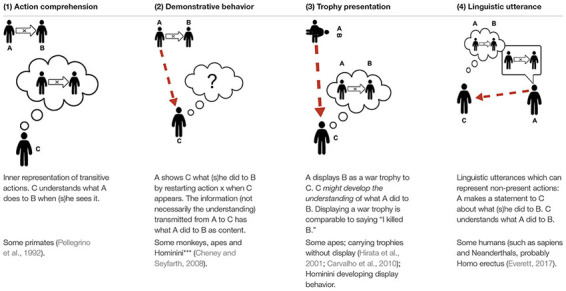

A = agent and sender, B = patient, C = receiver. Left column: primate cognition; the two middle columns show two different scenarios of indexical signaling: in column 2 the patient is a living creature, in column 3 an inanimate object. A is doing something to B (designated by the arrow with the x directed from A to B). A is informing C about the action (s)he did to B (designated by the red arrow with the broken line from A to C). ***Tribe including humans and their ancestors after the split from chimpanzees [cf. (Science, 2012, p. 538)].

### The Triangle Situation

In most primate societies the reproductive success of males depends on their position in the social hierarchy (de Waal, 1982). This puts hierarchy detection under selective pressure: on the one hand, individuals should avoid unnecessary hierarchical fights, and on the other hand, they need to engage in fights they are likely to win; otherwise they would fall in status. Due to its immense selective pressure and the complexity of factors contributing to hierarchy beyond physical strength [such as kin, character (e.g., aggressiveness), alliances, and rivalry] it seems likely that various cognitive abilities evolved for hierarchy detection. This would suggest that individuals get increasingly sensitive to social information. In this situation it might be beneficial not only to interpret such social signals but also to produce them, for instance, by displaying high status, alliances, or other social relations beneficial to communicate. This would likewise put under selective pressure the cognitive abilities that restrict such display behavior.

Now, let us imagine the following scenario as an example of the integration of indexical signs into communication: A defeats B in a hierarchical fight. After the fight has been completed, C enters the scene. C can probably infer intuitively the immediate past from its indexes: maybe B is wounded and behaving subserviently to A, who is puffed up and behaving dominantly. However, in cases where the outcome is less obvious it could be beneficial for A to display the outcome of the fight for at least three possible reasons: (a) to prevent an attack from C in the future (b) in case C is a desirable mate who might be attracted by A’s dominance, or (c) in case C is any conspecific who might otherwise benefit A by recognizing A’s status.

One way for A to demonstrate dominance would be to re-start the fight, which A would probably begin with a threatening gesture. Threatening gestures sometimes mimic fighting maneuvers. Animals that bite often bare their teeth; they also often scream or puff themselves up. In the described scenario, the threatening gesture (or chasing behavior if B flees)—by A toward B—transmits information about the past hierarchical fight that just took place, and it thereby displays hierarchical information to C (Table 1, column 2). This transmission of information connects a past event (what A did to B) with future behavior of C toward A, thereby we need not assume that C or A have a conscious representation of the past event; though restarting the fight with a threating gesture or actually attacking the opponent might be interpreted by an external observer as declaring “I defeated B” because both have a similar function. First the competence to fulfill the function develops [cf. (Dennett, 2017)] and only later the conscious representation of the past event. In this scenario, the information drawn from a complex social interaction provides the functional equivalent for the audience of understanding a proposition, though it probably misses crucial cognitive qualities that understanding something as a declaration would imply.

In this function-first approach to intentionality (aboutness), a complex social constellation works similarly to a cognitive system. In the earliest and simple form of this system, information *about something* is transmitted and processed without the cognition of a proposition: no symbolic thought, no concept of representation, no meta-cognition, nor even an intention to communicate. If C merely follows an algorithm such as “never fight against the winner of a fight, if you have not defeated the loser first,” then A’s behavior (restarting the fight) could be beneficial, without A having a concept of C’s mind and the intention to transmit a proposition (what A did to B) and without C having any belief about A’s past.

Obviously, this is only one of the possible scenarios in which indexical signs could have played a role in the cognitive development of our pre-human ancestors. I chose it because anything concerning hierarchy and sexual selection could have had a strong impact on evolutionary development, since both are essential for reproduction among primates in multi-male/multi-female groups. However, the example is introduced only to illustrate a broader principle.

### Limitation of the Triangle Situation

We have argued that a threatening gesture performed by A against B in the sight of C could be understood as a functional equivalent of a declaration by A to C. Declarations imply that they have truth values. This would mean that the threatening gesture becomes the kind of truth bearer which can be evaluated as true or false. The truth-maker in this scenario is the reaction of B. If B returns the threatening gesture aggressively, the relevant implication of the threatening gesture is falsified; if B, in contrast, retreats submissively, that implication is verified.

Although this scenario might put some cognitive abilities under selective pressure, there are at least three reasons to believe that this scenario is not a direct precursor of language:

(1) For full interpretation of a declaration the distinction between representation and reality has to be understood. Under these circumstances this essential semantic function could not have evolved. For this distinction to develop there must be cases in which the representation (what is expressed) and the reality (what is the case) could theoretically fail to correspond (whereas, in the case described above, the information drawn from the situation—including the reaction of B—cannot be disputed).
(2) Even an individual able to process language would probably perceive and understand this scenario intuitively and would absorb more information than could be expressed in a statement about the outcome of a fight, because what could be observed would be much richer than any linguistic expression. To reduce the past action to a proposition could thus be a disadvantage.
(3) A threatening gesture directed at one individual communicating something to another individual is a behavior we find in apes and monkeys [e.g., with baboons (Cheney and Seyfarth, 2008)] without them developing language-like behavior.

These objections would apply to most, if not all, scenarios in which a living creature and their behavior play the role of the patient. If conspecifics play the role of the patient, the truth of the implication of the gesture (indicating the past action) depends on reaction of the conspecifics, which cannot be completely controlled by the sender. Under these circumstances, the essential semantic function—the distinction between representation and reality—could not have evolved, which is a necessary prerequisite for interpreting anything as a fully-fledged declaration. It seems to be more likely that the binary value of truth emerges in cases in which the truth of a statement can be debated. For these reasons we shall reevaluate the trophy scenario. This will bring us to the most important part of this essay: the thesis that declaration emerged as a negation of an implicit negation in a dispute over truth in form of adding a simple mimetic gesture as the affirmation of the proposition the trophy display implies.

### Reviewing Trophy Display

We previously adopted a rather wide definition of what constitutes a trophy, making the hunting or war trophy only an example. We argued that if an individual displays an indexical object a syntax-like conceptual structure emerges: the presenter of the trophy constitutes the agent, the trophy represents the patient, and the verb (e.g., killing) is implied by the state of the patient. If a single mimetic gesture were to emerge it would be embedded in an implied syntax-like structure (for the relation between a physical and conceptual structure, see text Footnote 4). By integrating a directed mimetic gesture that represents the action into the spatial context, thereby marking the thematic roles (agent and patient), a simple form of declaration is expressed. This would be in line with the well-established idea that language evolved as sign language (Rizzolatti and Arbib, 1998; Corballis, 2002; Arbib, 2005). We also said before that it is unclear why anyone would introduce such a gesture, since it would not add any information to the scenario.

The suggestion I would like to discuss is that the explicit proposition (the adding of the mimetic gesture) would be the *rejection* of a somehow-expressed doubt about the implication of the trophy display. This is to say, the trophy displayer could naturally attempt to make such a gesture, if prompted to do so by someone else’s *rejection* or doubt of the presenter’s apparently desired implication.

Imagine a trophy display scenario including two hominin parties with different implicit interests: for one party it would be beneficial that the implicit statement (that the presenter of the trophy did in fact make the kill) is true and for the other party it would be beneficial if that were not true. Neither would need an explicit understanding in declarative form to have these implicit conflicting interests. Multiple observers would provide stronger motivation for the expression of doubts (whether the presenter made the kill), and their denial by the presenter, than situations in which only one onlooker were present.

To summarize the suggested scenario: (1) the presenter displays a trophy, (2) at least one onlooker understands the implication of the trophy display apparently desired by the presenter, (3) the receiver rejects the implication of the display (beneficial to the sender and not beneficial to the onlooker) by expressing disbelief or ignoring the trophy display, (4) the presenter of the trophy perceives and recognizes the rejection, and (5) the presenter expresses a declaration by adding a mimetic gesture to the trophy display in order to oppose the receiver’s rejection (the sign of disbelief or ignorance). The presenter addresses with the gesture the minds of all those present.

Nevertheless, mimetic gestures could arise for other reasons. If it is correct that action comprehension consists of two parts, mimesis and the suppression of the mimesis (internalization)—as Rizzolatti et al. (2001) suggest—then unintentional mimesis would be more primitive than action understanding. In some cases, a memory could trigger an involuntary mimetic gesture (especially in a young individual, whose inhibitory cognitive mechanisms are not fully yet developed). If such a gesture could improve the impact of presenting a trophy, it could develop. However, this would not be a declaration in a full sense for two reasons. Firstly, there would be no *intention* to communicate in this case, and secondly, the binary cognitive form of the propositional content is missing; this is to say it is not an explicit declaration because the sender does not comprehend the potential denial of the gesture’s implication. But here, again, a signal of disbelief could be expressed by a member of the audience and, as a reaction, the mimetic gesture would be affirmatively expressed. In either case this would constitute a full-fledged declaration as *the negation of a negation*^15^.

The point to be made here can be formulated as a paradox: *the concept of binarity is constituted in the negation of a value that is the product of the process of negation itself.* This is to say, expressing the explicit sentence in a simple form of spontaneous sign language is the *negation of a negation* (based on a rejection of a rejection) of the implied declaration of indexical signaling. Here, again, the concrete scenarios outlined above shall be understood as examples for illustrating a broader idea: The dispute over truth creates the binary structure underlying any declaration, which is the potential for dispute inherent in the concept of truth.

## Summary and Alternatives

### Alternatives and Evaluation

This is how I have argued so far: To express a declaration means to understand that it can be denied. External denial in a social situation is cognitively simpler than inner questioning. This is why I suggested that the first declarations occurred in social situations as the *negation of a negation*. Before the first declaration is expressed, no other declaration can exist, by definition. Thus, what is negated cannot be a declaration. This is to say that something that is not a declaration is treated as such (by denying it). This could have been an indexical signal, for instance a trophy displayed, or the display behavior could also include an involuntarily mimetic gesture triggered by a memory—expressed without the intention to make a statement. In the second case—in which the mimetic gesture emerges spontaneously because the presenter’s memory has been triggered during the display act—it would look like a declaration to us, however, it would not be a full-blown declarative sentence, because it would miss the binary cognitive form that is based on the understanding that the expression itself could be denied and also would lack the intention to communicate.

While I would accept both alternatives as different potential pathway, I would consider internal alternatives extremely unlikely—such as someone questioning themselves about what they ought to believe, without any social interaction. In this case, the propositional attitude would develop as self-reflection. To attack this Cartesian view [cf. (Ferretti and Adornetti, 2014] is one aim of this essay.

My hypothesis is compatible with many other suggestions: as soon as a simple language has developed it might have been used for many different purposes, including exchanging information about displaced resources (Bickerton and Szathmáry, 2011), coordinating actions by sharing intentions (Tomasello, 2008), teaching children (Fitch, 2004), communicating about the future and common goals (Gärdenfors, 2013) and for social grooming to build trust and social bonds as the basis of mating and cooperation (Dunbar, 2011a). The theory is also compatible with the idea that language evolved in the framework of sexual selection (Miller, 2000), which can include esthetic qualities of linguistic expression and thereby could explain the development of precise articulation, large lexicon, complex syntax and pleasant voice (which might have been selected for in a runaway process).

### The Suggested Solution to the Three Problems

In the beginning of this essay, I introduced three problems concerning language evolution: (1) to suggest the right kind of selective pressure, (2) the two problems of *irreducible complexity* in regard to declarative sentences, (3) the *cooperation paradox* (that language needs cooperation and human cooperation probably relies on gossip).

(1) I presented a scenario in which the speaker gains a reproductive advantage. Since speakers talk about themselves, they might influence the mental states of others in a beneficial way, which could affect social hierarchy and sexual selection.

(2) On the one hand, declarations require a minimum syntactic structure (an n-valence verb and n arguments, cf. Introduction), and on the other hand, they have truth values, which can be considered a meta-statement about the declaration which at the same time is implied in the declaration itself (cf. last subsection of the introduction).

The first aspect of the problem could be solved by our suggestion that an implied syntax-like structure emerges in displaying an indexical object (trophy). Here the whole situation works as an information-processing apparatus connecting a past behavior with future reproductive benefits. It is now quite a small step to add a directed mimetic gesture signifying the verb marking the sematic roles, which would turn the trophy display behavior into a simple sentence in non-conventional sign language. My solution for the second aspect of the problem of irreducible complexity is that the binary structure of declarations stems from conflicts of interest, as stated above.

In both suggested solutions, the higher order structure (*syntax* and *truth value* compared to linguistic elements and declarations) is founded in physical incorporating elements (body of the displayer, displayed object) and social situations (binary conflict of interest). In the first case syntax emerges as a relationship between objects before language begins and then becomes *internalized.* In the case of declaration and truth value the process is more complex. While an internalist might say that the cognition of truth values is suggested by the conflicts of perspective inherent in the situation, an externalist might suggest the following view: the binary form exists as the objective conflict of interest of individuals or parties about whether something is true. By addressing the beliefs of the others, the implicit conflict turns into a dispute over truth, in which declarations play an essential role and thereby gain their binary form.

(3) By postulating that the first declarations were expressed in the context of trophy displays we also solve the problem of how lying is prevented and how the truthfulness of a declaration emerging in a world without language could be in most cases ensured. We do not need to assume that a kind of *cooperative principle* [cf. (Grice, 1975] needed to be in place *before* language could begin to evolve. In turn, I suggest that the cooperative principle developed much later based on circulating stories about individuals, which might include whether an individual can be trusted to fulfill the cooperative principle, such as by speaking the truth. By this means our scenario explains both the evolution of language and the development of a social structure in which it becomes an *evolutionarily stable strategy* to cooperate.

My evolutionary hypothesis presented in this paper includes an empirically testable claim: We first evaluate whether a statement is beneficial for us, and (a few hundred milliseconds) later ask ourselves how we could justify the beneficial statement or the denial of a disadvantageous one. My proposition is thereby consistent with the *argumentative theory* by Mercier and Sperber (2011) and similar suggestions [such as (Wright, 1995; Haidt, 2012)]. However, this does not preclude epistemic progress, which develops historically not only through the technical advance of cultural techniques and scientific instruments, but also as the development of epistemic norms.

## Conclusion

In this essay I have argued that truth values are of social origin. The central hypothesis of this essay is that the first fully fledged declarations were *negations of negations* (based on rejections of rejections)^16^. The main idea is that explicit (propositional) beliefs did not develop for instrumental reasons, such as enhancing cognition by, for instance, reflecting step by step on the solution of a technical problem or decision making, but for addressing beliefs in others^17^. Thinking in language would be internalized speech—in contrast to Chomsky’s view in which speech is externalized thought [so called I-language (Chomsky, 1965)]. Accepting this view, language evolution begins as a symbolic communication about a past behavior of the communicator which addresses the mental states of conspecifics and thereby influences the reproductive rank of the communicator. This is to say, the hypothesis presented in this paper suggests that language began as virtue signaling and, so to say, as moral communication based on the capability to transform narratives about an individual into the image of their very nature. This hypothesis addresses both the problem of language evolution and the problems of human cooperation and also shows why early virtue signaling was reliable.

Early culture is not built on a biologically finished system, but the biological system adapts to social and cultural challenges both ontogenetically and phylogenetically (Richerson and Boyd, 2005). This would suggest that the dispute over truth could have catalyzed the evolution of the ability to understand the binary structure of truth.

We said that many animals address the mental states of other animals, including their conspecifics. However, there are two essential differences in the case of Hominini that distinguish it from most, if not all, other cases: firstly, in the Hominini case the mental states are addressed not by body parts (as in the case of the train of the peacock) nor by behavior (as in the case of the piping plover) but—in the case of trophy display—by *physical objects.* These natural signs which are finally used symbolically can be carried over a distance and work thereby as a *medium* for transmitting a past action. This is the beginning of the *media epistemic success story of human cognition*—from integrating indexical objects into communication (as discussed in this paper), counting with fingers (Kittler and von Heiseler, 2013), and totems symbolizing the group (Durkheim, 1912) to hieroglyphs, alphabetic writing (Ong, 1982), navigation, mapmaking, and the printing press, compass, telescope, microscope, computer, and the internet (Dennett, 2017).

Secondly, in the Hominini case the mental states that are addressed encode information about conspecifics (including the communicators), their actions and thereby their social status. One necessary condition for the emergence of the truth function is that brute force alone does not prevail, and that social hierarchy and female choice do not depend merely on physical strength. It has been suggested that the social structure of our ancestors changed when they started to use weapons, because at that point any more or less coordinated group could kill their alpha male in his sleep (Wrangham, 2019)^18^. Power, at that point, would depend on the mental states (representation or dispositions) of the relevant individuals in the group. The social structure would then rely to some extent on symbolic power and this symbolic power finally gains a self-referential logic (relying on what individuals believe others believe). The status of an individual no longer depends merely on their present, but also on what is *absent*: their past deeds. Here identity begins to be founded on narratives. Rank in the dominance hierarchy transforms into a social position, based on *reputation* (and coalitions) creating a new selective pressure resulting in the evolution of a distinctive human desire (Kojève, 1980): the desire for recognition [cf. (Hegel, 1807/1980]).

Accepting that culture plays a role in evolution—as has been shown even among non-human animals (Whitehead et al., 2019)—a trait being of natural and of social origin is not a contradiction (Laland, 2008). The idea that *the form of truth* is socially constructed and thus implies a normative attitude (individuals understand that some reasons are considered—by the relevant others—as good, others as bad) is thus compatible with *naturalism* (cf. e.g., Kornblith, 2002). Following this line of thought, history would not begin with the first civilizations or writing but with declarative sentences, maybe as early as 3 million years ago^19^. With the emergence of declarative sentences, propositional content—such as semantic knowledge and stories—could be transmitted between people of a culture and across generations, implying that humanness is a product of history, both phylogenetic and ontogenetic.

## Author Contributions

The author confirms being the sole contributor of this work and has approved it for publication.

## Conflict of Interest

The author declares that the research was conducted in the absence of any commercial or financial relationships that could be construed as a potential conflict of interest.

## Footnotes

1 I-language means *inner language*, however, the association with first-person singular and the Cartesian ego seems to be more than endured. For Chomsky language is I-language, while communication is secondary.

2 We understand a *proposition* as the *semantic content* of a declaration. A true proposition about the world is information about the world in a binary (and thus theoretically deniable) form. In this essay I use d*eclarative sentence* and *declaration* more or less interchangeably, although *declarations* include statements not grammatically well-formed as declarative sentences, such as for instance, often occurs in dialogs: A: “Did you wish your grandmother a happy birthday?”; B: “Forgot!” Here the same semantic content is expressed as in the declarative sentence “I forgot to wish my grandmother a happy birthday.”

3 The “ape test” as a principle for what makes a good theory of language evolution is explained in Gärdenfors’ essay *The Role of Cooperation in the Evolution of Protolanguage and Language* (Gärdenfors, 2013). Many scholars consider “the ape test” an important criterion (Számadó and Szathmáry, 2006).

4 This might be confusing because we are talking here about two different domains. What is done by the *brain* in the first case includes *an action of a situated speaker* in the second case. We might ask: “What about the brain of the speaker who performs the action?” The idea is that in the evolutionary process the action of the situated speaker precedes the full-fledged representation. From an *expanded mind* perspective, the meaning in the first case is encoded in the situation itself. An internalist might argue that *information* but not *meaning* can be processed in the environment and that any expression needs something to be expressed. An externalist might answer that understanding of actions is part of the motor system and is composed of imitation and suppression of the physical imitation (Fabbri-Destro and Rizzolatti, 2008). In this view the external processes are not just tools that prompted the mental processes, but they constitute them. This would suggest that communication precedes understanding (the inner presentation of what is communicated) and that competence precedes comprehension [cf. (Dennett, 2017)]. The external perspective is especially apt for evolutionary accounts, because competence and not cognition is selected for in the adaptive process.

5 For a criticism see Gould and Lewontin (1979), for the refutation of Gould’s criticism see Dawkins (1986), Maynard Smith (1988), Dennett (2017). As already noted, the goal of this paper is to explain neither the human mind nor a concrete cognitive process, but merely the *genetic foundation* of the ability to produce and understand declarative sentences and the distinction between representation and reality. We thereby embrace *methodical adaptationism*, which Godfrey-Smith (2001) defines as the idea that it is worth approaching biological systems from a perspective of adaptation, independent of any metaphysical claims.

6 While the change from land animals to sea animals happened several times [with seals, whales (descendants of hippos), sirenians (descendants of elephants), sea otters, and penguins] the successful development in the other direction only happened once (which makes it a good analogy for language).

7 Most linguistics theories do not use this classification and would probably make a categorical distinction between linguistic and non-linguistic imperatives or exclamations. A more common approach would be Searle’s taxonomy of illocutionary acts, which would complicate the argument unnecessarily (for reasons that cannot be explained in this footnote) and also would create confusion about the use of the term declarative (used by Searle in the meaning of a conventional performative act). The point to be made here is that human exclamations have a function comparable with animal cries expressing emotions like fear; and that chimpanzee gestures, which are expressed to influence the behavior of present conspecifics, are comparable to imperatives because they have a similar function. From this pragmatic perspective, humans use language for some of the functions fulfilled by animal communication, such as wooing, intimidating, submitting, threatening, begging; though nobody wants to deny that the structures of the utterances fulfilling the function differ fundamentally.

8 Apes trained in sign language never ask a question (which would imply understanding that someone else could know what they do not know) (Suddendorf, 2013b).

9 Cognitive simplicity (the ease with which something is understood by a cognitive system) is generally used as an argument for something being an evolutionary older structure (e.g., Buss, 2005) and that it is the form our mind is originally adapted to (e.g., Mercier and Sperber, 2011). If a trait first develops to comprehend a special form, this form should be more easily understood by the cognitive system in question than structures that are only understood later in evolutionary time.

10 I focus on the role of patient (something which is changed by being acted on) as opposed to other possible roles for syntactic objects, because I believe it describes the situations most likely to have served as vehicles for language evolution, as I describe further on.

11 It could be discussed whether technical images are mimetic and also produced somehow without intentions. The same would be true for reflections and shapes of shadows which on one hand are indexical signs and on the other can be mimetic in their shape.

12 Whether we shall call these mental states *beliefs* depends on how beliefs are defined: No, if we define beliefs as *representation* (Fodor, 1975; Fodor, 1981); yes, if we define them as *dispositions* (Marcus, 1990); maybe, if we attribute beliefs to creatures without language, like Lewis (1972), who believes that cognitive representation can have a map-like structure; no, if we believe, like Davidson (1982), that beings without language cannot have beliefs.

13 In most animals (inter)sexual selection is based on female mate choice (in general called “female choice”) (e.g., Majerus et al., 1986). The reasons why, in general, females are the choosing (selecting) sex [while males are, in general, chosen (sexually selected)] were explained by Trivers (1972), who developed a game theoretical model in which *parental investment* and *choosiness* correspond: the sex with the higher costs will choose their partner carefully and the chosen sex, the male, sometime develops traits according to the female preferences (Fisher, 1930). Please note, that I am not describing human courtship behavior but rather explaining how human traits could have developed from whatever existed before in the species from which we evolved. For this purpose, I assume that all uniquely human traits are absent, and look to the principles of mating we find in common among other primates living in multi-male/multi-female groups. These principles are dominance (intrasexual selection) and female choice (intersexual selection). In humans, however, males also choose their mates, especially when it comes to investments in their partners and their offspring (Buss and Schmitt, 1993). This might suggest that in the human case the trophy display theoretically could go in both directions. An evolutionary psychologist, on the contrary, might point out that only male chimpanzees hunt and engage in killing conspecifics from other groups and that in hunter-gatherer societies war and hunting big game is often left to males (although there are some small-scale societies in which females engage in hunting and war). Considering trophy display to some extent as part of sexual selection in the form of *costly signaling* (handicap principle) would provide evolutionary psychology with an evolutionary explanation why this might be the case.

14 That in general only males show such behavior suggests a sexual selection mechanism, but it does not mean that there are no other mechanisms at work.

15 Please note that “negation of negation” refers not to what is known in logic as *double negation elimination* (¬¬ *p*→*p*), but to a *social activity* of disagreeing. When two people, A and B, disagree by expressing alternating p and ¬ p (A: “p”; B: “¬ p”; A: “p!”; B: “¬ p!” …) it becomes clear that the same procedure (negation) can generate ¬ p from p as p from ¬ p. Why this intuitive assumption is true can be logically proven by double negation elimination, however, this logical grounding is not its cognitive source. The suggestion is that in the *activity of contradicting each other* the comprehension of the binary structure of the declarative sentence and its propositional content dawns.

16 In Spencer Brown’s (1969) line of thought: By processing a proposition three things are created: the proposition itself, its negation and the distinction between both. The distinction itself is founded on a transcendental form that allows to transform experience into statements. What is implicitly denied by a statement becomes the *excluded included other* (Spencer Brown, 1969). It is *excluded* because the declaration excludes the negation of itself and *included* because the declaration implies its negation (what it is not).

17 There are scholars, such as Habermas (1981), who would call both *instrumental*, which then would become a synonym for *self-interested*. From a game theoretical perspective following self-interest is to follow an *evolutionarily stable strategy*. To explain behavior by “true altruism” or “group selection” means giving up the possibility of understanding the structure of society (and thereby the possibility for a sound foundation of social sciences). The distinction we use here runs between instrumental and political intelligence [cf. (de Waal, 1982)].

18 In this case, culture (weapons) would have changed the social structure and the social structure would have changed selective pressures and thus the course of evolution. This would also explain the extraordinary pace of human evolution (Lahn et al., 2004).

19 Such developments start slowly and then accelerate. This would also be consistent with the rapid growth of the brains of our ancestors beginning about 2 million years ago. Hand axes only appear about 2.5 million years ago, but other weapons made of perishable materials could have appeared much earlier. Evidence for this is found in the adaptation of the wrist going back more than 4 million years (Lovejoy et al., 2009). I agree with Everett (2017) that Homo erectus is the best candidate to have developed language of a certain complexity and simple storytelling.

## References

[B1] Akmajian A. (1984). *Natural Language & Linguistic Theory. Sentence Types And The Form-Function Fit.* Berlin: Springer.

[B2] Alexander R. (1987). *The Biology of Moral Systems.* Hawthorne, NY: A. De Gruyter.

[B3] Alexander R. D. (2008). Evolution and human society. *Hum. Behav. Evol. Soc. Newslett.* 364 3127–3133.

[B4] Arbib M. A. (2005). From money-like action recognition to human language: an evolutionary framework for neurolinguistics. *Behav. Brain Sci.* 28 105–167. 16201457 10.1017/s0140525x05000038

[B5] Ardila A. (2016). The evolutionary concept of “preadaptation” applied to cognitive neurosciences. *Front. Neurosci.* 10:103 10.3389/fnins.2016.00103 PMC4794492 27013963

[B6] Austin J. L. (1962). *How to Do Things with Words.* Oxford: Clarendon Press.

[B7] Banta J. A. Richards C. L. (2018). Quantitative epigenetics and evolution. *Nature* 121 210–224. 10.1038/s41437-018-0114-x PMC6082842 29980793

[B8] Barclay P. (2010). *Reputation and the Evolution of Generous Behavior.* New York, NY: Nova Science Publishers.

[B9] Baron-Cohen S. (1999). “Evolution of a theory of mind?,” in *The Descent Of Mind: Psychological Perspectives On Hominid Evolution*, eds Corballis M. Lea S. (Oxford: Oxford University Press).

[B10] Behe M. J. (1996). *Darwin’s Black Box: Biochemical Challenge to Evolution.* New York, NY: Free Press.

[B11] Bickerton D. (2005). Language evolution: a brief guide for linguists. *Lingua* 117 510–526.

[B12] Bickerton D. (2009). *Adam’s Tongue.* New York, NY: Hill and Wang.

[B13] Bickerton D. (2010). “On two incompatible theories of language evolution,” in *The Evolution of Human Language: Biolinguistic Perspectives (Approaches to the Evolution of Language)*, eds Larson R. D. Yamakido H. (Cambridge: Cambridge University Press).

[B14] Bickerton D. Szathmáry E. (2011). Confrontational scavenging as a possible source for language and cooperation. *BMC Evol. Biol.* 11:art261. 10.1186/1471-2148-11-261 PMC3188516 21933413

[B15] Blumberg M. Freeman J. Robinson S. (2009). *Oxford Handbook of Developmental Behavioral Neuroscience: Epigenetics, Evolution, and Behavior.* Oxford: Oxford University Press.

[B16] Buss D. (2005). *The Handbook of Evolutionary Psychology.* New Jersey: Wiley.

[B17] Buss D. M. Schmitt D. P. (1993). Sexual strategies theory: an evolutionary perspective on human mating. *Psychol. Rev.* 100 204–232. 8483982 10.1037/0033-295x.100.2.204

[B18] Byrne R. W. Whiten A. (1988). *Machiavellian Intelligence: Social Expertise And The Evolution Of Intellect In Monkeys, Apes And Humans.* Cambridge: Cambridge University Press.

[B19] Carvalho S. Yamanashi Y. Yamakoshi G. Matsuzawa T. (2010). *Bird In The Hand: Bossou Chimpanzees (Pan Troglodytes) Capture West African Wood-Owls (Ciccaba Woodfordi) But Not To Eat.* London: Pan Afr News.

[B20] Cheney D. L. Seyfarth R. M. (2008). *Baboon Metaphysics: The Evolution of a Social Mind.* Chicago: University of Chicago Press.

[B21] Chomsky N. (1956). Three models for the description of language. *IRE Trans. Inform. Theory* 2 113–124.

[B22] Chomsky N. (1965). *Aspects of the Theory Of Syntax.* Cambridge MA: MIT Press.

[B23] Chomsky N. (1966). *Cartesian Linguistics: A Chapter in the History of Rationalist Thought.* New York, NY: Harper & Row.

[B24] Chomsky N. (1993). *A Minimalist Program For Linguistic Theory. MIT Occasional Papers In Linguistics No. 1.* Cambridge, MA: MIT.

[B25] Chomsky N. (1999). *Derivation by Phase.* Cambridge, MA: MIT Press.

[B26] Chomsky N. (2006). *Language and Mind.* Cambridge: Cambridge University Press.

[B27] Chomsky N. (2010). “Some simple evo devo theses: how true might they be for language,” in *The Evolution of Human Language. Biolinguistic Perspectives*, eds Larson R. K. Déprez V. Yamakido H. (Cambridge: Cambridge University Press), 45–62.

[B28] Clark A. (2016). *Surfing Uncertainty: Prediction, Action, and the Embodied Mind.* Oxford: Oxford University Press.

[B29] Clark J. Montellano M. Hopson J. Hernndez R. Fastovsky D. (1994). “An early or middle jurassic tetrapod assemblage from the la boca formation, Northeastern Mexico,” in *In the Shadow of the Dinosaurs: Early Mesozoic Tetrapods*, eds Fraser N. Sues H.-D. (Cambridge: Cambridge Univ. Press), 295–302.

[B30] Copi I. M. Cohen C. McMahon K. (2014). *Introduction to Logic*, 14th Edn, Abingdon: Rutledge.

[B31] Corballis M. (2002). *From Hand to Mouth. The Origins of Language.* Princeton: Princeton University Press.

[B32] Corballis M. (2016). The evolution of language: sharing our mental lives. *J. Neuro.* 43 120–132.

[B33] Cuskley C. Little H. Ravignani A. McCrohon M. F. Verhoef T. (2018). “The Evolution Of Language,” in *Proceedings Of The 12th International Conference On The Evolution Of Language (Evolang12)*, Torun.

[B34] Darwin C. (1859). *On the Origin of Species by Means of Natural Selection, or the Preservation of Favoured Races in the Struggle for Life.* London: John Murray. PMC5184128 30164232

[B35] Davidson D. (1982). Rational animals. *Dialectica* 36 317–327.

[B36] Dawkins R. (1986). *The Blind Watchmaker: Why the Evidence of Evolution Reveals a Universe without Design.* New York, NY: Norton.

[B37] Dawkins R. Krebs J. R. (1978). “Animal signals: information or manipulation,” in *Behavioural Ecology: An Evolutionary Approach*, eds Krebs J. R. Davies N. B. (Oxford: Blackwell), 282–309.

[B38] de Waal F. (1982). *Chimpanzee Politics.* Baltimore: John Hopkins University Press.

[B39] Deacon T. W. (1997). *The Symbolic Species: The Co-Evolution of Language and the Brain.* New York, NY: W.W. Norton & Co.

[B40] Dennett D. (2017). *From Bacteria to Bach and Back.* London: Penguin.

[B41] Dobzhansky T. (1937). *Genetics and the Origin of Species.* Irvine, CA: National Academy of Sciences Beckman Center.

[B42] Dunbar R. (2011a). “Gossip and the social origins of language,” in *Oxford Handbook of Language Evolution*, eds Tallerman M. Gibson S. (Oxford: Oxford University Press).

[B43] Dunbar R. (2011b). The social brain meets neuroimaging. *Trends Cogn. Sci.* 16 102–122. 22177800 10.1016/j.tics.2011.11.013

[B44] Durkheim É (1912). *Les Formes Élémentaires De La Vie Religieuse.* Paris: Presses Universitaires de France.

[B45] Everett D. (2017). *How Language Began: The Story of Humanity’s Greatest Invention.* New York, NY: Liveright.

[B46] Fabbri-Destro M. Rizzolatti G. (2008). Mirror neurons and mirror systems in monkeys and humans. *Int. Union Physiol. Sci.* 23 171–179. 10.1152/physiol.00004.2008 18556470

[B47] Ferretti F. Adornetti I. (2014). Against linguistic cartesianism: toward a naturalistic model. *Lang. Commun.* 37 29–39.

[B48] Ferretti F. Adornetti I. Chiera A. Cosentino E. Nicchiarelli S. (2018). Introduction: origin and evolution of language—an interdisciplinary perspective. *Topoi* 37 219–234.

[B49] Fisher R. A. (1930). *The Genetical Theory of Natural Selection.* Oxford: Clarendon.

[B50] Fitch T. W. (2010). *The Evolution of Language.* Cambridge: Cambridge University Press.

[B51] Fitch W. T. (2004). “Kin selection and mother tongues: a neglected component in language evolution,” in *Evolution of Communication Systems: A Comparative Approach*, eds Griebel U. Oller D. K. (Cambridge, MA: MIT Press), 275–296.

[B52] Fodor J. (1975). *The Language of Thought.* Cincinnati: Crowell.

[B53] Fodor J. A. (1981). *Representations.* Cambridge, MA: MIT Press.

[B54] Frege G. (1892). Über sinn und bedeutung. *Zeitschrift Philos. Philos. Kritik* 100 25–50.

[B55] Friederici A. D. (2020). Hierarchy processing in human neurobiology: how specific is it?. *Philos. Trans. R. Soc. Lond. Ser. B* 375:20180391 10.1098/rstb.2018.0391 PMC6895560 31735144

[B56] Gärdenfors P. (2013). “The role of cooperation in the evolution of protolanguage and language,” in *Evolution of Mind, Brain, and Culture*, eds Hatfield G. V. Pittman H. (Pennsylvania: University of Pennsylvania Press), 193–216.

[B57] Gintis H. (2011). “Gene–culture coevolution and the nature of human sociality,” in *The Alphabet and the Brain*, eds de Kerckhove D. Lumsden C. J. (Berlin: Springer). 10.1098/rstb.2010.0310 PMC3048999 21320901

[B58] Godfrey-Smith P. (2001). “Three kinds of adaptationism,” in *Adaptationism and Optimality*, eds Orzack S. H. Sober E. (New York, NY: Cambridge University Press), 335–357.

[B59] Goldin-Meadow S. (1978). A study in human capacities. *Science* 200 649–651. 17812701 10.1126/science.200.4342.649

[B60] Goodall J. (1977). Infant killing and cannibalism in free-living chimpanzees. *Folia Primatol.* 28 259–282. 564321 10.1159/000155817

[B61] Gould S. J. Eldredge N. (1972). “Punctuated equilibria: an alternative to phyletic gradualism,” in *Models in Paleobiology*, ed. Schopf T. (San Francisco: Freeman Cooper & Co), 82–115.

[B62] Gould S. J. Lewontin R. (1979). The Spandrels of San Marco and the Panglossian Paradigm: A Critique of the adaptationist programme. *Proc. R. Soc. B* 205 581–598. 10.1098/rspb.1979.0086 42062

[B63] Gould S. J. Vrba E. S. (1982). Exaptation – a missing term in the science of form. *Paleobiology* 8 4–15.

[B64] Grice H. P. (1975). “Logic and conversation,” in *Speech Acts*, eds Cole I. P. Morgan J. L. (New York, NY: Academic Press), 41–58.

[B65] Grice P. H. (1957). Meaning. *Philos. Rev.* 66 377–388.

[B66] Habermas J. (1981). *Theorie des Kommunikativen Handelns.* Frankfurt: Suhrkamp.

[B67] Haidt J. (2012). *The Righteous Mind: Why Good People Are Divided by Politics and Religion.* New York, NY: Pantheon.

[B68] Hamilton W. D. (1964). The genetical evolution of social behaviour I & II. *J. Theor. Biol.* 7 1–52. 5875341 10.1016/0022-5193(64)90038-4

[B69] Hare B. Call J. Tomasello M. (2001). Do chimpanzees know what conspecifics know and do not know? *Anim. Behav.* 61 139–151. 11170704 10.1006/anbe.2000.1518

[B70] Hauser M. D. Charles R. Berwick C. Tattersall I. Ryan M. J. Watumull J. (2014). The mystery of language evolution. *Front. Psychol.* 5:401 10.3389/fpsyg.2014.00401 PMC4019876 24847300

[B71] Hegel G. W. (1807/1980). *Phänomenologie des Geistes.* Hamburg: Rheinisch-Westfälische Akademie der Wissenschaften.

[B72] Herrmann E. Call J. Hernàndez-Lloreda M. V. Hare B. Tomasello M. (2007). Humans have evolved specialized skills of social cognition: the cultural intelligence hypothesis. *Science* 317 1360–1366. 17823346 10.1126/science.1146282

[B73] Hirata S. Yamakoshi G. Fujita S. Ohashi M. (2001). Capturing and toying with hyraxes (Dendrohyrax dorsalis) by wild chimpanzees (Pan troglodytes) at Bossou Guinea. *Am. J. Primatol.* 53 93–97. 11170171 10.1002/1098-2345(200102)53:2<93::AID-AJP5>3.0.CO;2-X

[B74] Hobaiter C. Byrne R. W. (2014). The meanings of chimpanzee gestures. *Curr. Biol.* 24 1596–1600. 24998524 10.1016/j.cub.2014.05.066

[B75] Humphrey N. (1976). “The social function of intellect,” in *Growing Points in Ethology*, eds Bateson P. Hinde R. (Cambridge: Cambridge University Press), 303–317.

[B76] Hurford J. (1999). “The evolution of language and languages,” in *The Evolution of Culture*, ed. Dunbar R. E. (Edinburgh: Edinburgh University Press), 173–193.

[B77] Jablonka E. Lamb M. J. (2008). Soft inheritance: challenging the modern synthesis. *Genet. Mol.* 31 389–395.

[B78] Janvier P. Clément G. (2010). Palaeontology: muddy tetrapod origins. *Nature* 463 40–41. 20054387 10.1038/463040a

[B79] Janzen T. (2017). Composite utterances in a signed language: topic constructions and perspective-taking in ASL. *Cogn. Linguist.* 28:121.

[B80] Kellogg W. N. (1931). Humanizing the ape. *Psychol. Rev.* 38 160–176.

[B81] Kittler F. von Heiseler T. N. (2013). *Flaschenpost an die Zukunft.* Berlin: Kadmos.

[B82] Kojève A. (1980). *Introduction to the Reading of Hegel: Lectures on the Phenomenology of Spirit.* New York, NY: Cornell University Press.

[B83] Kornblith H. (2002). *Knowledge and its Place In Nature.* Oxford: Oxford University Press.

[B84] Krebs D. (1998). “The evolution of moral behavior,” in *Handbook Of Evolutionary Psychology: Ideas, Issues, and Application*, eds Crawford C. Krebs D. (Abingdon: Psychology Press).

[B85] Lahn B. Dorus S. Vallender E. J. Evans P. D. Anderson J. R. Gilbert S. L. (2004). Accelerated evolution of nervous system genes in the origin of homo sapiens. *Cell* 119 1027–1040. 15620360 10.1016/j.cell.2004.11.040

[B86] Laland K. N. (2008). Exploring gene–culture interactions: insights from handedness, sexual selection and niche-construction case studies. *Philos. Trans. R. Soc. B Biol. Sci.* 363 3577–3589. 10.1098/rstb.2008.0132 PMC2607340 18799415

[B87] Lerner J. Tetlock P. (2003). “Bridging Individual, interpersonal, and institutional approaches to judgment and decision making: the impact of accountability on cognitive bias,” in *Emerging Perspectives on Judgment and Decision Research*, eds Schneider S. Shanteau J. (Cambridge: Cambridge University Press), 431–457.

[B88] Lewis D. (1972). Psychophysical and theoretical identifications. *Austr. J. Philos.* 50:1972.

[B89] Lovejoy C. O. Simpson S. W. White T. D. Asfaw B. Suwa G. (2009). Careful climbing in the miocene: the forelimbs of ardipithecus ramidus and humans are primitive. *Science* 326 e1–e8. 19810196

[B90] Lucchesi J. C. (2019). *Epigenetics, Nuclear Organization & Gene Function.* Oxford: Oxford University Press.

[B91] Majerus M. E. N. O’Donald P. Kearns P. W. E. Ireland H. (1986). Genetics and evolution of female choice. *Nature* 321 164–167. 10.1038/321164a0 21227769

[B92] Marcus R. B. (1990). Some revisionary proposals about belief and believing. *Philos. Phenomenol. Res.* 50 133–153.

[B93] Maynard Smith J. (1964). Group selection and kin selection. *Nature* 201 1145–1147.

[B94] Maynard Smith J. (1988). *Did Darwin Get It Right? Essays on Games, Sex And Evolution.* London: Penguin.

[B95] Maynard Smith J. (1994). Must reliable signals always be costly. *Anim. Behav.* 47 1115–1120.

[B96] Maynard Smith J. Szathmáry E. (1995). *The Major Transitions in Evolution.* New York, NY: Oxford University Press.

[B97] Mayr E. (2001). *What Evolution Is.* New York, NY: Basic Books.

[B98] Mercier H. Sperber D. (2011). Why do humans reason? Arguments for an argumentative theory. *Behav. Brain Sci.* 34 57–111. 21447233 10.1017/S0140525X10000968

[B99] Miller G. (2000). *The Mating Mind: How Sexual Choice Shaped The Evolution Of Human Nature.* London: Heineman.

[B100] Mitani J. C. Watts D. P. (2001). Why do chimpanzees hunt and share meat? *Anim. Behav.* 61:91.

[B101] Newman S. A. Bhat R. (2009). Dynamical patterning modules: a “pattern language” for development and evolution of multicellular form. *Intern. J. Dev. Biol.* 53 693–705. 10.1387/ijdb.072481sn 19378259

[B102] Niedźwiedzki G. Szrek P. Narkiewicz K. Narkiewicz M. Ahlberg P. E. (2010). Tetrapod trackways from the early middle devonian period of Poland. *Nature* 463 43–48. 20054388 10.1038/nature08623

[B103] Ong W. J. (1982). *Orality and literacy: The technologizing of the Word.* New York, NY: Methuen.

[B104] Peirce C. S. (1931). *Collected Papers of Charles Sanders Peirce.* Cambridge, MA: Belknap Press.

[B105] Pellegrino G. Fadiga L. Fogassi L. Gallese V. Rizzolatti G. (1992). Understanding motor events: a neurophysiological study. *Exp. Brain Res.* 91 176–180. 1301372 10.1007/BF00230027

[B106] Pigliucci M. Müller G. (2010). *Evolution – the Extended Synthesis.* Cambridge, MA: MIT Press.

[B107] Pinker S. (1995). *The Language Instinct: How the Mind Creates Language.* New York, NY: HarperCollins.

[B108] Pinker S. Jackendoff R. (2004). The faculty of language: what’s special about it. *Cognition* 95 201–236. 10.1016/j.cognition.2004.08.004 15694646

[B109] Pinker S. Jackendoff R. (2005). The nature of the language faculty and its implications for evolution of language (Reply to Fitch, Hauser, and Chomsky). *Cognition* 97 211–225. 10.1016/j.cognition.2005.02.005 16112662

[B110] Power C. (1998). “Old wives’ tales: the gossip hypothesis and the reliability of cheap signals,” in *Approaches to the Evolution Of Language*, eds Hurford J. R. Studdert-Kennedy M. Knigh C. (Cambridge: Cambridge University Press), 111–129.

[B111] Richerson P. Boyd R. (2005). *Not By Genes Alone: How Culture Transformed Human Evolution.* Chicago: University of Chicago Press.

[B112] Rizzolatti G. Arbib M. (1998). Language within our grasp. *Trends Neurosci.* 21 188–194. 9610880 10.1016/s0166-2236(98)01260-0

[B113] Rizzolatti G. Bucciono G. (2005). “The mirror neuron system and its role in imitation and language,” in *From Monkey Brain to Human Brain*, eds Dehaene S. Duhamel J. E. (Cambridge, MA: MIT Press), 213–233.

[B114] Rizzolatti G. Fogassi L. Gallese V. (2001). Neurophysiological mechanisms underlying the understanding and imitation of action. *Nat. Rev. Neurosci.* 2 661–670. 11533734 10.1038/35090060

[B115] Saussure F. D. (1916). *Cours de Linguistique Générale.* Geneva: Charles Bally.

[B116] Science (2012). Band 336, Nr. 6081. Science.

[B117] Spencer Brown G. (1969). *Laws of Form.* London: Georg Allen.

[B118] Sperber D. Baumard N. (2012). Moral reputation: an evolutionary and cognitive perspective. *Mind Lang.* 27 495–518. 10.1111/mila.12000

[B119] Suddendorf T. (2013a). Mental time travel: continuities and discontinuities. *Trends Cogn. Sci.* 17 151–152. 23453751 10.1016/j.tics.2013.01.011

[B120] Suddendorf T. (2013b). *The Gap: The Science of What Separates Us from Other Animals.* New York, NY: Basic Books.

[B121] Számadó S. Szathmáry E. (2006). Competing selective scenarios for the emergence of natural language. *Trends Ecol. Evol.* 21 555–561. 16828925 10.1016/j.tree.2006.06.021

[B122] Tallerman M. Gibson K. R. (2013). *The Oxford Handbook of Language Evolution.* Oxford: Oxford University Press.

[B123] Taylor D. L. Jackson A. U. Narisu N. Hemani G. Erdos M. R. Chines P. S. (2019). Integrative analysis of gene expression, DNA methylation, physiological traits, and genetic variation in human skeletal muscle. *PNAS* 116 10883–10888. 31076557 10.1073/pnas.1814263116 PMC6561151

[B124] Tesnière L. (2015/1966). *Elements of Structural Syntax.* Amsterdam: John Benjamins.

[B125] Tinbergen N. (1963). On aims and methods of Ethology. *Zeitschrift für Tierpsychologie* 20 410–433.

[B126] Tomasello M. (2008). *The Origins of Human Communication.* London: MIT Press.

[B127] Tomasello M. (2019). *Becoming Human A Theory of Ontogeny.* Harvard: Harvard Press.

[B128] Trivers R. (1972). “Parental investment and sexual selection,” in *Sexual Selection and the Descent of Man*, ed. Campbell B. (Chicago: Springer), 136–179.

[B129] Verkuyl H. J. Swart H. D. Hout A. V. (2006). *Perspectives on Aspect.* New York, NY: Springer.

[B130] von Heiseler T. N. (2019). Syntax of testimony: indexical objects, syntax, and language or how to tell a story without words. *Front. Psychol.* 10:477. 10.3389/fpsyg.2019.00477 30967805 PMC6438894

[B131] Wacewicz S. Żywiczyński P. (2018). Language origins: Fitness consequences, platform of trust, cooperation, and turn-taking. *Interact. Stud.* 19 167–182.

[B132] Whitehead H. Laland K. N. Rendell L. Thorogood R. Whiten A. (2019). The reach of gene–culture coevolution in animals, nature communication. *Nat. Commun.* 10:2405. 10.1038/s41467-019-10293-y PMC6546714 31160560

[B133] Williams G. (1966). *Adaptation and Natural Selection.* Princeton: Princeton University Press.

[B134] Williams M. (2004). “Is knowledge a natural phenomenon?,” in *The Externalist Challenge*, ed. Schantz R. (Berlin: De Gruyter), 2–193.

[B135] Wilson D. S. (2006). “Human groups as adaptive units: toward a permanent consensus,” in *The Innate Mind: Culture and Cognition*, eds Carruthers P. Laurence S. Stich S. (Oxford: Oxford University Press).

[B136] Wilson D. S. Wilson E. O. (2008). Evolution for the good of the group. *Am. Sci.* 96 380–389.

[B137] Wrangham R. (2019). *The Goodness Paradox: The Strange Relationship Between Virtue and Violence in Human Evolution.* New York, NY: Pantheon.

[B138] Wright R. C. (1995). *The Moral Animal: Evolutionary Psychology And Everyday Life.* New York, NY: Vintage Books.

[B139] Zahavi A. (1975). Mate selection – a selection for a handicap. *J. Theor. Biol.* 53 205–214. 1195756 10.1016/0022-5193(75)90111-3

[B140] Zahavi A. (1993). The fallacy of conventional signaling. *Philso. Trans. R. Soc. Lond. B* 338 227–230. 10.1098/rstb.1993.0061 8101657

